# Integrated high‐throughput phenomics and transcriptomics uncover the transcriptional mechanisms underlying rice responses to elevated CO_2_ concentration and cadmium stress

**DOI:** 10.1002/imo2.70100

**Published:** 2026-05-20

**Authors:** Weijun Guo, Hanlin Liu, Dongwei Li, Shang Xie, Yichao Mao, Liwen Yang, Cong Li, Weixuan Wang, Jian Tian, Xiaofeng Gu, Li Pu

**Affiliations:** ^1^ Biotechnology Research Institute, Chinese Academy of Agricultural Science Beijing China; ^2^ Institute of Animal Science, Chinese Academy of Agricultural Science Beijing China

**Keywords:** cadmium, elevated carbon dioxide, integrative network, phenomics, superior allele, transcriptomics

## Abstract

Developing crop varieties with reduced cadmium (Cd) accumulation under elevated carbon dioxide (eCO_2_) is a major global challenge. Identifying hub genes and elucidating the transcriptional mechanisms governing Cd stress and eCO_2_ responses requires integrated, multi‐tissue omics approaches. Here, we conducted high‐throughput phenotyping of the rice cultivar Nipponbare across six key developmental stages under Cd stress and three eCO_2_ concentrations. Image‐based traits revealed shared and distinct phenotypic responses to these stresses. Multi‐tissue transcriptome analyses showed that the differentially expressed genes (DEGs) and hormone‐related genes exhibited a similar expression pattern under Cd and eCO_2_. We further constructed an integrative co‐expression network and identified five stress‐responsive subnetworks. Among these, *Multiple Stress Responsive Gene 3* (*OsMSR3*) emerged as a hub gene, with its haplotype 2 (Hap2) frequency decreasing from low‐ to high‐latitude regions and from southern to northeastern China, mirroring the geographic distribution of Cd‐contaminated soils. Collectively, our study reveals a transcriptional regulatory network underlying rice responses to Cd and eCO_2_ stresses, providing mechanistic insights and valuable genetic resources for future molecular breeding of low‐Cd accumulating rice varieties adapted to constant rising CO_2_ levels.

## Highlights

- Acquisition of high‐throughput phenomics covering six key growth stages of rice cultivar Nipponbare under eCO_2_ and Cd stress.
- Differentially expressed genes and hormone‐related genes exhibit a similar expression pattern under Cd and eCO_2_ using multi‐tissue transcriptome analysis.
- We constructed an integrative co‐expression network and identified five stress‐responsive subnetworks.
- Haplotype 2 frequency of hub gene *OsMSR3* decreases from low‐ to high‐latitude regions and from southern to northeastern China, mirroring the geographic distribution of Cd‐contaminated soils.

## Graphical abstract

Developing crop varieties with reduced cadmium (Cd) accumulation under elevated carbon dioxide (eCO_2_) requires hub gene identification and elucidation of its transcriptional mechanisms. Here, we acquired high‐throughput phenotyping of the rice cultivar Nipponbare across six key developmental stages under Cd stress and three eCO_2_ concentrations. An integrative network based on multi‐tissue transcriptome was successfully constructed, and five key stress‐responsive subnetworks were identified, in which hub gene *OsMSR3*′s haplotype 2 frequency was decreased from low‐ to high‐latitude regions and from southern to northeastern China, mirroring the geographic distribution of Cd‐contaminated soils. Our findings offer novel insight into the transcriptional regulation of rice in response to eCO_2_ and Cd stress, providing valuable genetic resources for molecular breeding of low‐Cd accumulating rice varieties adapted to constant rising CO_2_ levels in the future.

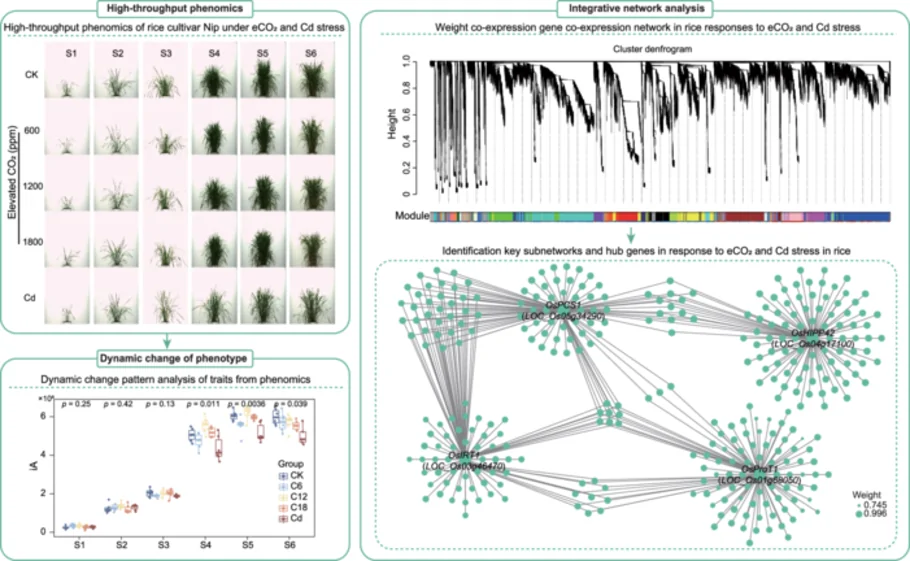

## 1 INTRODUCTION

Meeting the food and nutritional demands of a global population projected to reach 9 billion by the mid‐21st century will require an approximately 60% increase in agricultural production, despite mounting challenges from land degradation, water scarcity, and environmental pollution [1]. Climate change has intensified the frequency and severity of both biotic and abiotic stresses, substantially reducing crop yields, threatening food security, and undermining progress toward sustainable development goals. In particular, anthropogenic stresses such as elevated carbon dioxide (eCO_2_) and heavy metal contamination have profoundly affected agricultural systems [1]. Atmospheric CO_2_ stands at approximately 415 μmol mol^−1^ [2], and is projected to rise to 550−650 μmol mol^−1^ by mid‐century and to ~ 800 μmol mol^−1^ by the end of the 21st century according to a model prediction result [3]. Crops grown under eCO_2_ conditions often exhibit 3−17% reductions in protein, iron, and zinc contents, posing serious risks to global human nutrition [3]. Meanwhile, cadmium (Cd) is a highly toxic heavy metal that accumulates in agricultural soils and transfers along the soil‐plant‐human continuum, causing severe environmental damage and health risk [4, 5]. In plants, Cd is taken up by roots and transported to shoots via specific transporter proteins, several of which have been identified [5]. Thus, understanding how eCO_2_ influences Cd accumulation is critical for sustainable agricultural production.

Rice (*Oryza sativa* L.), a staple crop comprising the *japonica* and *indica* subspecies, exhibits a particularly high capacity for Cd uptake and accumulation [6]. Typically, *indica* rice accumulates more Cd in grains and shoots than *japonica* rice [7]. Notably, a single allelic variation in the *DNR1* (*DULL NITROGEN RESPONSE1*), a regulator of nitrate‐use efficiency, drives divergent responses to eCO_2_ between these subspecies [8]. Several studies have examined the interactive effects of eCO_2_ and Cd‐contaminated soils on plants [9, 10, 11, 12, 13, 14, 15]. In rice, eCO_2_ alters different effects on essential and non‐essential amino acid concentrations in grains [9] and increases Cd concentrations in both shoots and grains when grown in Cd‐contaminated soils [11]. Other studies have similarly evaluated Cd uptake by rice under eCO_2_ in Cd‐contaminated paddy soils [16]. In contrast, some reports indicate no significant changes in Cd content in cereals or legumes under eCO_2_ [15]. However, the dynamic phenotypic responses across key developmental stages and the tissue‐specific transcriptional regulatory networks underlying rice responses to Cd and eCO_2_ remain poorly understood.

Plants have evolved diverse detoxification mechanisms to cope with Cd stress [5]. In rice, several transporter proteins, including OsNRAMP5 and OsCd1, have been characterized [17, 18, 19, 20, 21, 22, 23]. OsNRAMP5, localized to the plasma membrane, is a major transporter mediating Cd uptake, and loss‐of‐function mutants exhibit up to 90% reductions in Cd accumulation [17, 18]. OsCd1, a member of the major facilitator superfamily, also contributes to Cd transport, with *oscd1* knockout mutants displaying lower Cd concentrations in roots and shoots than in the wild‐type plants [19]. Natural variation in *OsCd1* partly explains the divergence in grain Cd accumulation between *japonica* and *indica* subspecies [19]. In addition, several transcriptional regulators involved in Cd response have been identified [24, 25, 26, 27, 28, 29, 30, 31]. Among them, *OsMSR3* (also known as *OsHSP18.0‐CI*), a class I sHSP family heat shock protein, is induced by Cd stress, and the transgenic *Arabidopsis* expressing this gene exhibits enhanced Cd tolerance, suggesting a positive regulatory role in Cd stress responses [24]. Phytohormones, including abscisic acid (ABA), jasmonic acid (JA), and salicylic acid (SA), also participate in plant Cd stress responses [32, 33, 34]. Although these studies provide valuable insights, the dynamic regulation of rice under Cd stress and the mechanistic interactions with eCO_2_ remain unclear.

Although the interactive effects of eCO_2_ and Cd‐contaminated soils on plant phenotypes have been investigated [9, 10, 11, 12, 13, 14, 15], the transcriptional mechanisms underlying these responses remain unresolved. To address this gap, we conducted eCO_2_ experiments on rice cultivar Nipponbare under three eCO_2_ concentrations using a free‐air CO_2_ enrichment (FACE) system and performed a Cd stress experiment using a high‐throughput phenotyping (HTP) platform. We integrated high‐throughput phenomics with multi‐tissue transcriptome profiling to construct an integrative regulatory network and elucidate the transcriptional mechanisms governing rice responses to Cd and eCO₂ stress.

## 2 RESULTS

### Dynamic phenotypic changes in rice under Cd and eCO_2_ conditions

To characterize the phenotypic variation in rice under Cd and eCO_2_ stress, we conducted experiments using a high‐throughput automatic phenotyping platform (HTP) [35]. A total of 31 time‐point phenomic datasets were collected across six key developmental stages‐seedling growth, tillering, elongation, booting, flowering, and maturation stage (S1 to S6) for the rice cultivar Nipponbare under Cd stress, three eCO_2_ concentrations, and normal condition (control, CK). Dynamic patterns of phenotypic traits across these stages were systematically analyzed (Figure 1A).

**Figure 1:**
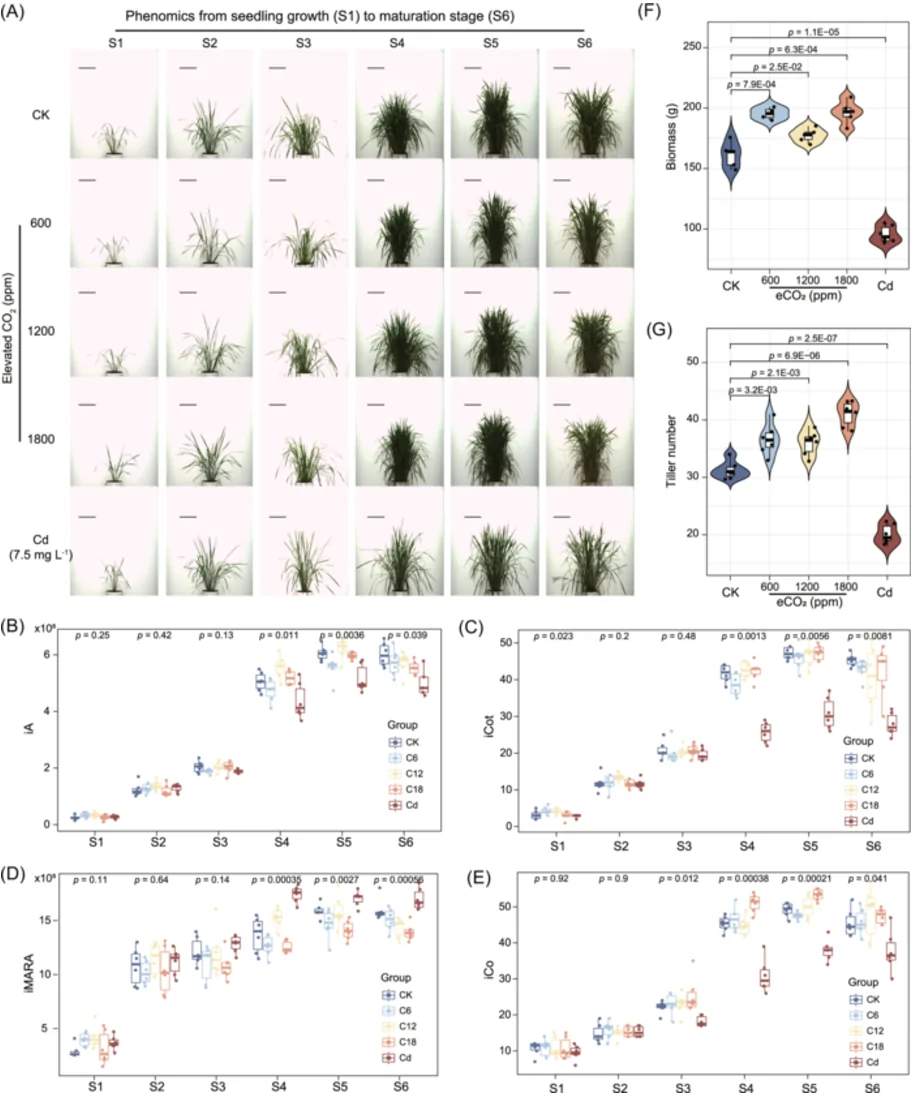
High‐throughput phenomics of rice under elevated carbon dioxide (eCO_2_) concentration and cadmium (Cd) stress. (A) The representative images of rice subspecies *japonica* cultivar Nipponbare at six key growth stages spanning from the seedling growth stage (S1) to maturation stage (S6) under three elevated carbon dioxide (eCO_2_) concentration degrees, cadmium (Cd) stress conditions, and the blank group (referred to as the CK) from a high‐throughput phenomics platform. Each group contained six rice plants. The detailed description of each treatment was seen in the method section. Bar = 25 cm. Dynamic growth trends of image‐based area (i‐A) (B) and compactness of top view image (i‐Cot) (C) of rice under eCO_2_ and Cd stress, which showed similar decreased trends to the control samples at the maturation stage (S6). The image‐based min area rectangle area (iMARA) (D) and compactness of front view image (E) showed a contrary tendency that iMARA was decreased under both three eCO_2_ degree conditions and increased under Cd stress, but image‐based compactness (iCo) was increased under eCO_2_ and decreased under Cd stress at the S6 stage compared with the control samples under normal conditions. The biomass (F) and tiller number (G) at the maturation stage (S6) were significantly increased under eCO_2_ and significantly decreased under Cd stress compared to the normal conditions. Statistical significances were determined by Kruskal–Wallis methods (B−E) and two‐tailed Student's *t*‐test (F, G), and the *p*‐value < 0.05 represents a significant difference. Abbreviation: S1 to S6 represent the seedling growth, tillering, elongation, booting, flowering, and maturation stages, respectively; CK, blank group; eCO_2_, elevated carbon dioxide; Cd, cadmium. The three eCO_2_ groups were referred to as C6, C12, and C18, corresponding to being treated with 600, 1200, and 1800 additional μmol mol^−1^ CO_2_ more than the CK. i‐A, image‐based area; iCo, image‐based compactness; i‐Cot, compactness of top view image; iMARA, image‐based min area rectangle area.

Fifty‐four image‐based traits (i‐traits) were measured per plant at each time point, of which seven representative i‐traits were selected for detailed comparison from S1 to S6: image‐based area (i‐A), compactness top (i‐Cot), minimum area rectangle area (i‐MARA), compactness (i‐Co), area top (i‐At), second moment principal axis large norm (i‐MAPLA), and minimum enclosing circle diameter (i‐MECD) (Figure 1B−D and Figure S1). The i‐A representing the rice plant area in the front‐view image, increased rapidly from S3 to S4 and then approached a plateau (Figure 1B), while the i‐Cot, reflecting top‐view compactness, exhibited a similar trend (Figure 1C). Both i‐A and i‐Cot emerged significantly different at S4 and persisted through later stages among these five groups (*p* = 0.011, 0.0036, and 0.039 for S4, S5, and S6, respectively) (Figure 1B,C). Although plants under Cd stress showed the slowest increase in i‐A and i‐Co compared with control and eCO₂‐treated plants, both Cd and eCO_2_ ultimately led to reduced i‐A at maturation (Figure 1B,C). The i‐At (image‐based area top) and i‐MPALA followed dynamic patterns similar to those of i‐A and i‐Cot (Figure S1A,B).

To further dissect the differential effects of Cd and eCO_2_, we examined i‐traits exhibiting distinct trends (Figure 1D,E and Figure S1C). The i‐MARA increased rapidly at S2 and gradually stabilized thereafter; at S6, i‐MARA values were higher under Cd stress but lower under eCO_2_ relative to controls (Figure 1D). The i‐MECD (image‐based min enclosing circle diameter) displayed trends consistent with i‐MARA (Figure S1C). In contrast, i‐Co (image‐based compactness) increased sharply from S3 to S4, and was lower at S6 under Cd stress, but higher under eCO_2_, exhibiting an inverse pattern relative to i‐MARA (Figure 1E).

Finally, we assessed two key agronomic traits‐biomass and tiller number‐through manual measurements (Figure 1F,G). Both traits increased significantly under all three eCO_2_ groups (biomass: *p* = 7.9E‐04, 2.5E‐02, 6.3E‐04; tiller number: *p* = 3.2E‐03, 2.1E‐03, and 6.9E‐06 for C6, C12, and C18 groups, respectively), but decreased significantly under Cd stress compared with the controls (*p* = 1.1E‐05 and 2.5E‐07, respectively) (Figure 1F,G). Together, these results reveal both shared and distinct phenotypic responses to Cd and eCO_2_, indicating that these i‐traits are effective indicators for quantifying stress intensity in rice.

### Multi‐tissue transcriptomes reveal shared transcriptional functions in response to Cd and eCO_2_

To investigate the transcriptional regulatory differences under Cd and eCO_2_, we selected two representative developmental stages: the elongation stage (S3; ES) and the maturation stage (S6; MS), representing the vegetative and reproductive phases, respectively. Leaves and stems were sampled at both stages, and young panicles were collected at MS for RNA‐seq analysis. Two independent biological replicates were sequenced, resulting in ~342.5 Gb of raw data (Table S1). On average, 88.23% of reads were uniquely mapped and used for gene expression quantification (Table S1). Principal component analysis was performed on FPKM (Fragments per kilobase per million mapped reads)‐normalized expression values (Figure 2A), and Pearson correlation coefficients confirmed high concordance between biological replicates (Figure S2).

**Figure 2:**
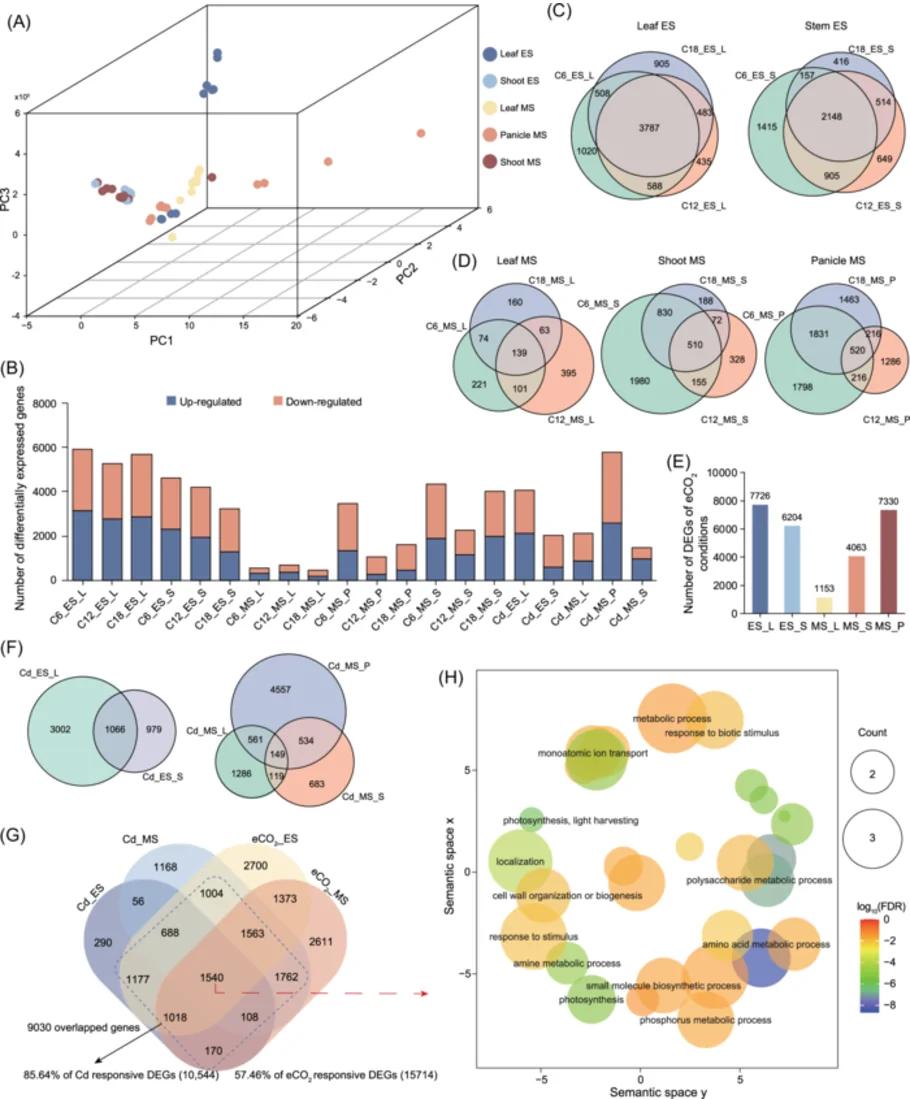
Tissue‐specific transcriptomic profile of rice in response to eCO_2_ and Cd stress. (A) Principal component analysis of the transcriptome of two representative growth stages (elongation and maturation stages (S3 and S6), hereafer referred as ES and MS, respectively) of rice under elevated CO_2_ (eCO_2_) concentration and cadmium (Cd) stress, which was grouped by tissue and growth stage combination that these samples were divided into leaf sample at ES (Leaf_ES), stem sample at ES (Stem_ES), leaf sample at MS (Leaf_MS), stem sample at MS (Stem_MS), and young panicle sample at MS (Panicle_MS). (B) Number of differentially expressed genes (DEGs) identified in leaf, stem, and young panicle tissues under different stresses. These DEGs were divided into up‐ and down‐regulated expression that were compared with the blank group (CK). The three C6, C12, and C18 (eCO_2_ groups) represent that the rice plants were treated with 600, 1200, and 1800 additional μmol mol^−1^ CO_2_ more than the control group. L, S, and P represent the leaves, stem, and young panicles. (C and D) Comparisons of DEGs of different tissues at ES (C) and MS (D) under three different degrees of eCO_2_ conditions. (E) The number of DEGs under eCO_2_ conditions, where the DEGs under different eCO_2_ conditions in the same group were merged. (F) Comparisons of DEGs of different tissues at ES and MS under Cd stress. (G) Venn diagrams showing the overlap of DEGs under eCO_2_ and Cd stress, in which DEGs were grouped by a combination of growth stage and stress, which were divided into four categories: eCO_2__ES, eCO_2__MS, Cd_ES, and Cd_MS. (H) Gene Ontology (GO) enrichment results of 1540 overlapped genes in the four groups in G, which were significantly enriched in “response to stimulus,” “photosynthesis, light harvesting,” and “metabolic process,” etc., and the detailed description was seen in Table S3. ES and MS, elongation and maturation stages; eCO_2_, elevated carbon dioxide; Cd, cadmium; DEGs, differentially expressed genes; GO, gene ontology.

The differentially expressed genes (DEGs) were identified using gene count matrices, with a threshold of ≥ two fold change and false discovery rate (FDR) < 0.05 relative to the corresponding controls (Figure 2B). More DEGs were detected at ES than at MS, and leaf samples at MS under eCO_2_ exhibited the fewest DEGs (Figure 2B). DEGs under three CO_2_ concentrations (C6, C12, and C18) were classified into five groups based on tissue and developmental stage: leaf_ES, stem_ES, leaf_MS, stem_MS, and panicle_MS. A total of 3787, 2148, 139, 510, and 520 were shared across the three eCO_2_ levels in these respective groups (Figure 2C,D). To further assess tissue‐ and stage‐specific responses, DEGs from all three eCO_2_ treatments were merged into five categories: ES leaf (ES_L), ES stem (ES_S), MS leaf (MS_L), MS stem (MS_S), and MS panicle (MS_P) (Figure 2E). DEG numbers declined in both leaves and stems at MS compared with ES, whereas young panicles exhibited the highest number of DEGs at MS (Figure 2E).

Overall, 15,714 DEGs were identified and grouped into five expression clusters (C1 to C5) (Figure S3A). Cluster 1 genes were highly expressed in leaf samples and significantly enriched in “photosynthesis (FDR = 1.1E‐13),” etc. Cluster 2 genes were highly expressed in the leaves at both ES and MS and in the stem at MS, and were significantly enriched “defense response (FDR = 5.7E‐08),” “response to stress (FDR = 2.2E‐03),” and “cell death (FDR = 5.7E‐08)”. Cluster 3 genes were predominantly expressed in panicles and stems at MS and were significantly enriched in “regulation of nitrogen compound metabolic process (FDR = 7.3E‐16)”. Cluster 4 and Cluster 5 genes were highly expressed in the stems at MS and ES, respectively, and were both significantly enriched in the bZIP (basic leucine zipper) transcription factor family members (*p* < 0.05) and also significantly enriched in “carbohydrate metabolic process (FDR = 7.5E‐15 and 8.5E‐14),” etc. (Figure S3B,C). These findings indicate that dynamic gene expression plays a central role in rice responses to eCO_2_.

We also examined DEGs under Cd stress at the same developmental stages, identifying 1219 and 286 shared DEGs in ES and MS, respectively (Figure 2F). To elucidate the functional roles of these DEGs, we performed Gene Ontology (GO) functional enrichment analyses for each group (Figures S4−S6). DEGs from leaves and stems under both Cd and eCO_2_ stress were significantly enriched (FDR < 0.05) in common terms, such as “secondary metabolic process” and “photosynthesis”; while DEGs in young panicles were significantly enriched in “cell growth” and “cell cycle” (Figures S4−S6), suggesting convergent transcriptional regulation under both stresses.

Strikingly, 85.64% (9030 of 10,544) DEGs in the Cd group overlapped with those in the eCO_2_ group, and 57.46% of the 15,714 eCO_2_‐responsive DEGs overlapped with Cd‐responsive genes. Among these, 1540 genes were differentially expressed in both Cd and eCO_2_ treatments at ES and MS (Figure 2G and Table S2). GO enrichment analysis of these shared DEGs revealed predominant involvement in “photosynthesis, light harvesting (FDR = 2.7E‐04),” “response to stimulus (FDR = 7.6E‐03),” and “carbohydrate metabolic process (FDR = 2.2E‐09)” (Figure 2H and Table S3).

In summary, rice exhibits tissue‐specific transcriptional responses to Cd and eCO_2_, with extensive overlap in DEGs between the two stresses, indicating that similar functional pathways are activated to mitigate Cd toxicity and adapt to elevated eCO_2_.

### Dynamic changes in hormone‐related genes under Cd and eCO_2_ conditions

Phytohormones, such as abscisic acid (ABA), jasmonic acid (JA), and salicylic acid (SA), are known to mediate plant responses to Cd stress [32, 33, 34]. To characterize broader hormonal regulation under Cd and eCO_2_ stress, we systematically examined hormone‐related genes across the transcriptome. In total, 682 expressed genes that are potentially involved in the regulatory pathways of eight major hormones‐auxin, SA, ethylene, ABA, brassinolide (BR), cytokinin (CK), JA, and gibberellic acid (GA)‐were identified from the transcriptome (Figure 3A−E and Figure S7, and Tables S4−S11). Based on Mapman annotations, these hormone‐related genes were classified into three functional categories: synthesis and degradation (C1), signal transduction (C2), and induced regulated responsive activated (C3).

**Figure 3:**
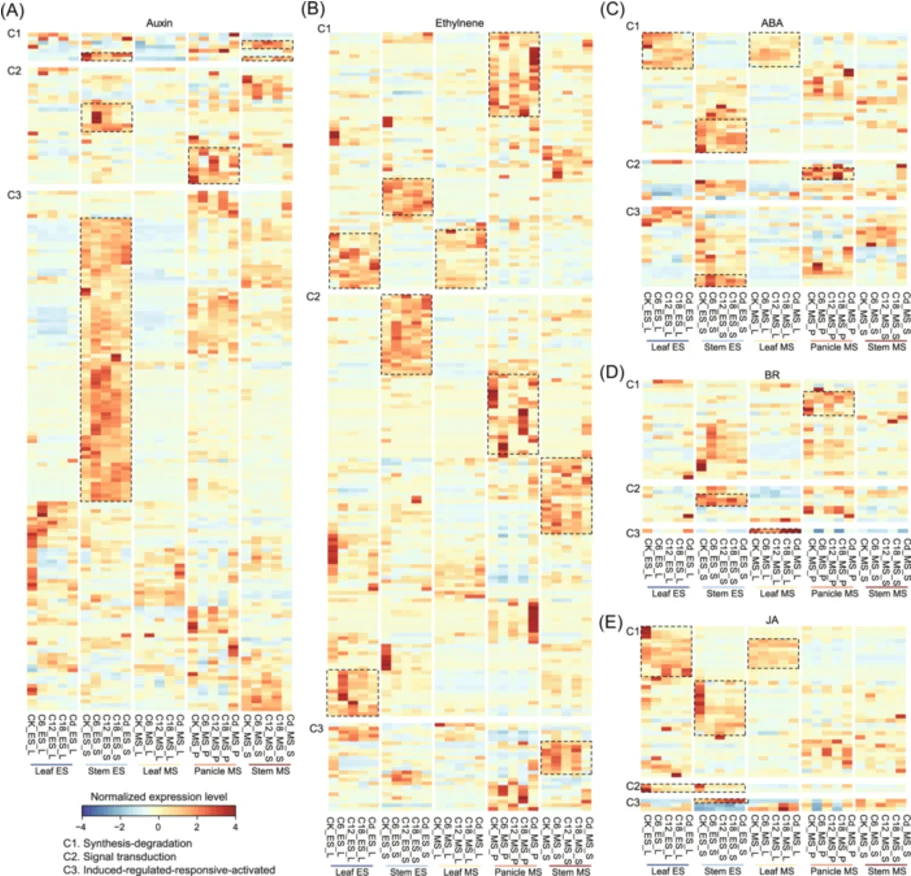
Dynamic expression patterns of hormone‐related genes. Normalized gene expression levels related to (A) auxin, (B) ethylene, (C) abscisic acid (ABA), (D) brassinolide (BR), and (E) jasmonic acid (JA) are shown. For each hormone, its genes are divided into three functional categories: (C1) synthesis degradation, (C2) signal transduction, and (C3) induced regulated responsive activated. Dotted rectangles represent these genes expressed in a tissue‐specific manner. ABA, abscisic acid; BR, brassinolide; JA, jasmonic acid.

Most of the hormone‐related genes exhibited strong tissue specificity under both Cd and eCO_2_ treatment (Figure 3 and Figure S7, dotted rectangles). For example, the C1 genes of SA and JA, the C2 genes of BR and CK, and the C3 genes of auxin and ABA were preferentially expressed in the stems at the elongation stage (ES) and displayed similar expression trends under Cd and eCO_2_ (Figure 3A−E and Figure S7A−C). The C1 genes of JA were specifically expressed in leaves at both ES and maturation stage (MS) and in stem at ES, among these, the *12‐oxo‐phytodienoic acid reductase gene* (*OsOPR1*), previously implicated Cd tolerance [36], was down‐regulated in both leaf and stem tissues under Cd and eCO_2_ groups (Figure 3E), suggesting shared hormonal regulation under both stresses.

Ethylene, which plays key roles in both stress responses and plant growth and development [37], displayed distinct regulatory patterns. We found that the C2 and C3 ethylene‐related genes were weakly expressed in leaves at MS, whereas C3 genes were specifically expressed in the stems at MS, exhibiting opposite responses to the two stresses: up‐regulation under eCO_2_ but down‐regulation under Cd stress (Figure 3B). These findings indicate divergent roles of ethylene signaling in response to Cd versus eCO_2_. Overall, hormone‐related genes showed strong tissue specificity, with subsets displaying concordant or contrasting expression patterns under Cd and eCO_2_, underscoring the complexity of hormonal regulation during stress adaptation.

### Construction of an integrative network in response to Cd and eCO_2_ in rice

To elucidate the transcriptional regulatory networks underlying rice responses to Cd and eCO_2_, we performed weighted gene co‐expression network analysis (WGCNA) using all expressed genes to construct a regulated network and conducted association analyses between gene modules and phenotypic traits (Figure 4A−C). A soft‐thresholding power of 17 was selected based on scale‐free topology and mean connectivity (Figure 4A). Gene clustering was performed using a dynamic tree cut algorithm on the topological overlap matrix, resulting in 31 co‐expression modules labeled by distinct colors (Figure 4B). Module eigengene (ME), representing the overall expression profiles of each module, was correlated with phenotypic traits under Cd and eCO_2_ stress (Figure 4C).

**Figure 4:**
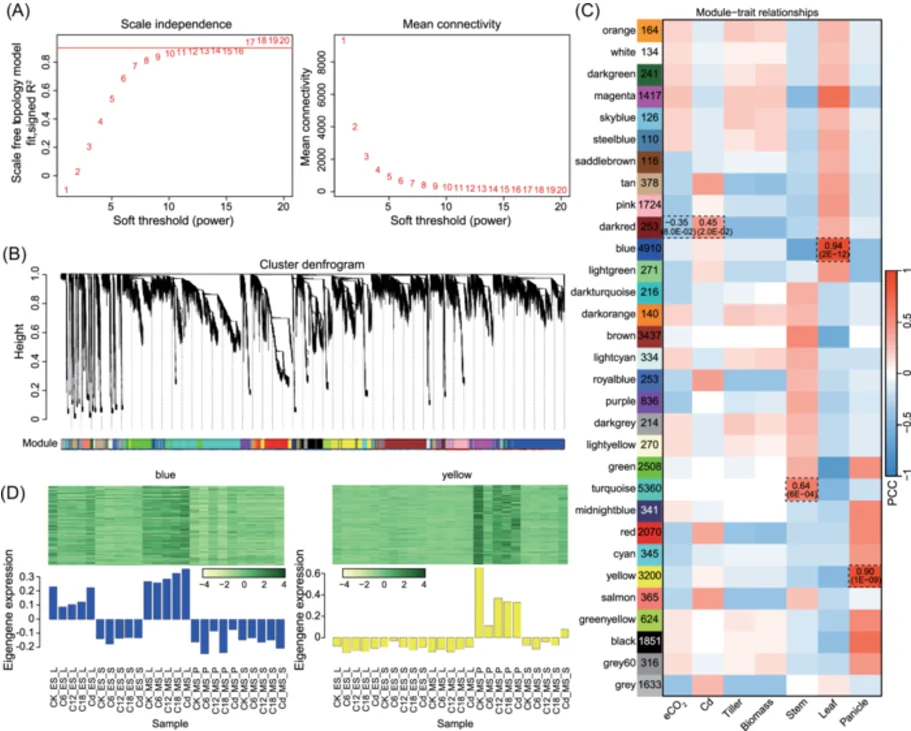
Weight gene co‐expression network analysis of multi‐tissue transcriptomes under eCO_2_ concentration and Cd stress. (A) The scale independence and mean connectivity at different soft thresholds, where the soft threshold value 17 was used for the next network construction. (B) The hierarchical cluster of all genes and the gene module partition. A total of 31 modules are labeled with colors. (C) The correlation analysis between modules and traits, where the correlation coefficient (ranging from −1 to 1) and the *p*‐value for the statistical significance were labeled in each group. The gene number in each module was labeled on the left side. Dotted rectangles represent tissue‐specific expressed genes. (D) Heatmap of blue and yellow modules, which are significantly positively associated with the leaf (*R* = 0.94, *p*‐value = 2E‐12) and panicle (*R* = 0.90, *p*‐value = 1E‐09), respectively, showed the expression profile of all genes in corresponding modules.

We focused on modules showing the strongest and most significant associations (*p *< 0.05). Consistent with tissue‐specific transcriptomic responses, several modules were strongly associated with individual tissues (Figures 2, 4, and 3). The turquoise, blue, and yellow modules comprising 5360, 4910, and 3200 genes, respectively, were significantly correlated with stem (*R* = 0.64, *p* = 6E‐04), leaf (*R* = 0.94, *p* = 2.0E‐12), and panicle (*R* = 0.90, *p* = 1.0E‐09) tissues (Figure 4C,D). Notably, the dark‐red module comprising 253 members showed a positive association with Cd stress (*R* = 0.45, *p* = 2.0E‐02), and a negative association with eCO_2_ stress (*R* = −0.35).

Within these modules, several known stress‐related genes were identified. In the yellow module, *OsCLT1*, encoding a chloroquine‐resistance transporter (CRT)‐like transporter involved in Cd stress response [25], was down‐regulated in ES leaf, ES stem, MS leaf, and MS panicle under Cd stress. The *small heat shock protein* (*OsHSP18.0‐CI*, also known as *OsMSR3*) gene, previously shown to enhance heavy metal tolerance when overexpressed in rice [38] was up‐regulated in the panicle and stem tissues at MS. GO enrichment analyses further supported functional relevance: blue module genes were significantly enriched in terms of “photosynthesis (FDR = 4.8E‐08)” and “response to stimulus (FDR = 7.1E‐08)”; yellow module genes in “regulation of primary metabolic process (FDR = 5.6E‐10)” and “protein metabolic process (*p* = 8.1E‐09)”; turquoise module genes in “response to oxidative stress (FDR = 1.4E‐05)” and “microtubule‐based process (FDR = 1.0E‐04)”; and dark‐red module genes in “cell death (FDR = 3.6E‐02)”, “response to stress (FDR = 3.6E‐02)”, and “defense response (FDR = 3.6E‐02)” (Figure S8 and Table S12). Together, these findings highlight key gene modules that likely play central roles in rice responses to Cd and eCO_2_ stress and provide valuable resources for dissecting transcriptional regulation under these conditions.

### Transcriptional regulatory subnetworks of rice respond to Cd and eCO_2_

By integrating known Cd‐ and eCO_2_‐responsive genes, we constructed hub‐centered regulatory subnetworks within the four key modules (Figure 5A−D). In total, five subnetworks encompassing nine previously reported genes were identified, including two subnetworks within the yellow module, and one each subnetwork in the blue, turquoise, and dark‐red modules (Figure 5 and Tables S13−S16).

**Figure 5:**
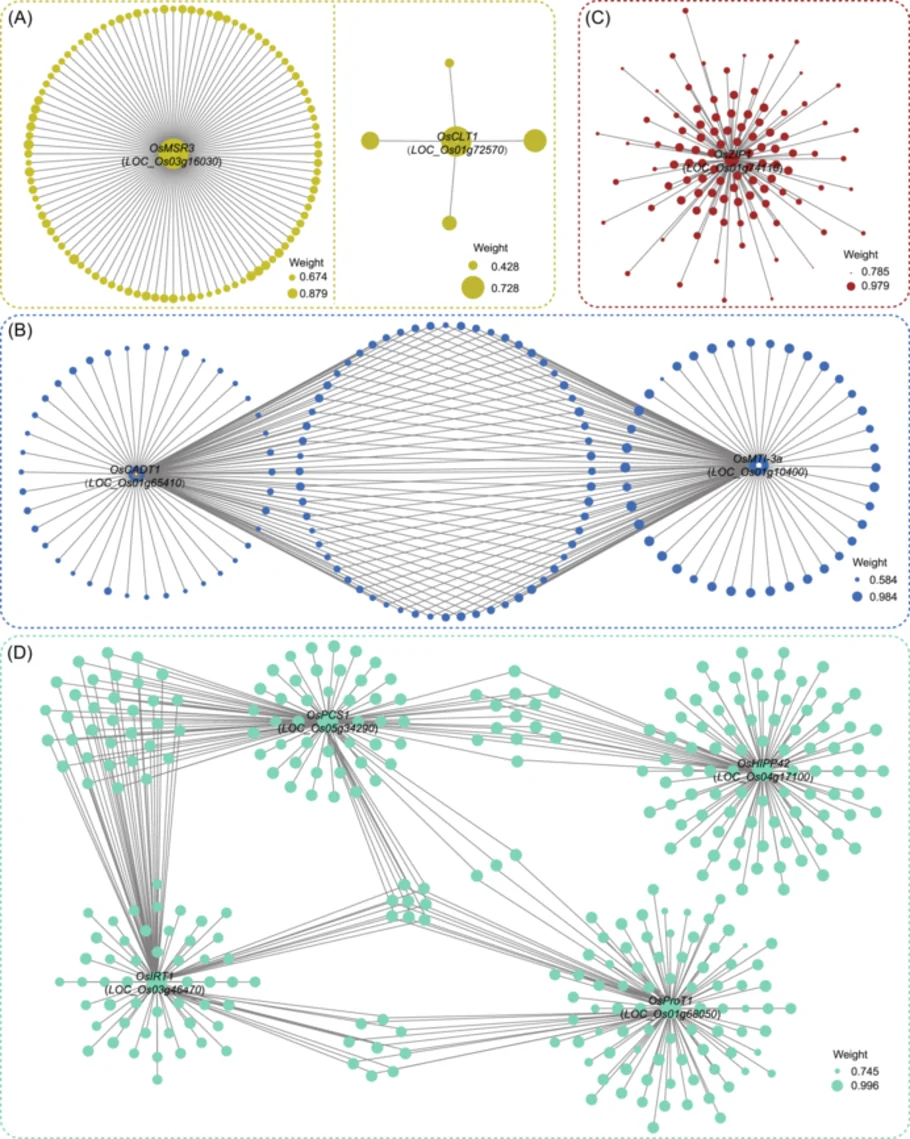
The putative gene regulatory networks in response to eCO_2_ concentration and Cd stress in rice. (A) Two hub subnetworks were identified from the yellow module that were significantly positively associated with the leaf. Two known genes, *OsMSR3* and *OsCLT1*, were the hub genes in corresponding modules. (B) The subnetwork identified from the blue model was significantly positively associated with the panicle. Two known genes, *OsCADT1* and *OsMTI‐3a*, were the hub genes that together regulated most of the target genes in the network. (C) Subnetwork identified from the dark red module that was significantly positively associated with the Cd stress (*R* = 0.45, *p*‐value = 2E‐02). The known gene *OsZIP1* was the hub gene in this network. (D) Subnetwork identified from the turquoise module that was significantly positively associated with the stem (*R* = 0.64, *p*‐value = 6E‐04). Four known genes, *OsPCS1*, *OsIRT1*, *OsProT1*, and *OsHIPP42*, were the hub genes, where *OsPCS1*, *OsIRT1*, and *OsProT1* shared the targeted genes, and *OsHIPP42* only shared the targeted genes with *OsPCS1*. The size of the circle represents the weight value of the gene in the subnetwork.

In the yellow module, well‐characterized Cd‐responsive genes, *OsMSR3* and *OsCLT1*, served as central hubs. *OsMSR3*, a class I small heat shock protein, is induced by Cd stress, and the transgenic *Arabidopsis* expressing this gene exhibits enhanced Cd tolerance, indicating its positive regulatory role in Cd stress responses [24]. We identified 100 genes that co‐expressed with *OsMSR3*, many of which may contribute to Cd tolerance (Figure 5A)*. OsCLT1*, encoding a CRT‐like transporter, is the causal gene of an arsenate [As(V)]‐sensitive rice mutant and plays a critical role in glutathione homeostasis, thereby influencing As and Cd detoxification; the *Osclt1* mutant is also hypersensitive to Cd stress [25]. Four genes were co‐expressed with *OsCLT1*, and this subnetwork showed no overlap with the *OsMSR3*‐centered network (Figure 5A). The yellow module was strongly associated with panicle tissue and exhibited high expression levels, indicating that these subnetworks may play key roles in Cd detoxification during reproductive development (Figure 4C,D).

In the blue module, associated with leaf tissue, we identified a subnetwork centered on two known Cd‐responsive genes: *type 3 metallothionein‐like gene* (*OsMTI‐3a*), a type 3 metallothionein‐like gene that confers heavy metal tolerance by mitigating reactive oxygen species (ROS), and *cadmium tolerant 1* (*OsCADT1*), whose mutation enhances Cd tolerance [26, 27]. Sixty genes were co‐expressed with these two hubs, suggesting coordinated regulation of leaf‐specific Cd responses (Figure 5B). In the dark‐red module, which was strongly associated with Cd stress, we identified an *OsZIP1*‐centered subnetwork comprising 100 target genes (Figure 5C). Finally, in the turquoise module associated with stem tissue, we identified a subnetwork containing four known metal‐related genes: *phytochelatin synthase* (*OsPCS1*) [28], *iron‐regulated metal transporter* (*OsIRT1*) [29], *proline transporter* (*OsProT1*) [30], and *heavy metal‐associated isoprenylated plant protein* (*OsHIPP42*) [31]. The *OsPCS1*, *OsIRT1*, and *OsProT1* shared multiple target genes, with eight targeted genes that were co‐regulated by all three genes, indicating similar transcriptional regulatory mechanisms in Cd stress responses. In contrast, *OsHIPP42* shared target genes only with *OsPCS1*, suggesting a more specific regulatory interaction (Figure 5D). Collectively, these hub‐centered subnetworks anchored by known Cd‐responsive genes provide a comprehensive framework for understanding the transcriptional mechanisms underlying rice responses to Cd and eCO_2_ stress and offer valuable genetic resources for breeding low‐Cd accumulating rice varieties adapted to elevated CO_2_ conditions.

### A natural *OsMSR3* variation may contribute to subspecies differences in rice Cd and eCO_2_ tolerance

To evaluate the potential application of the identified hub genes in natural populations, we selected *OsMSR3* as a model for single‐nucleotide polymorphism (SNP) discovery and haplotype analysis within the coding DNA sequence (CDS) using 3K Rice Genome database (RFGB) [39] and rice modern Chinese rice cultivars from the RiceAtlas database (see Methods) [40] (Figure 6A−G). Integrating phenomic i‐traits, we first assessed correlations between *OsMSR3* expression levels and key i‐traits. OsMSR3 expression was significantly negatively correlated with i‐A (*R* = −0.45, *p* = 4.5E‐02), i‐MARA (*R* = −0.56, *p* = 1.0E‐02), and i‐MECD (*R* = −0.50, *p* = 2.5E‐02), and showed negative associations with i‐At (*R* = −0.40, *p* = 7.7E‐02) and i‐MPALA (*R* = −0.43, *p* = 5.9E‐02) (Figure 6A), suggesting that *OsMSR3* may positively regulate rice tolerance to both the Cd and eCO_2_. Consistently, previous studies reported that overexpression of *OsMSR3* enhances the tolerance to heavy metal stress, including Cd, in rice [38].

**Figure 6:**
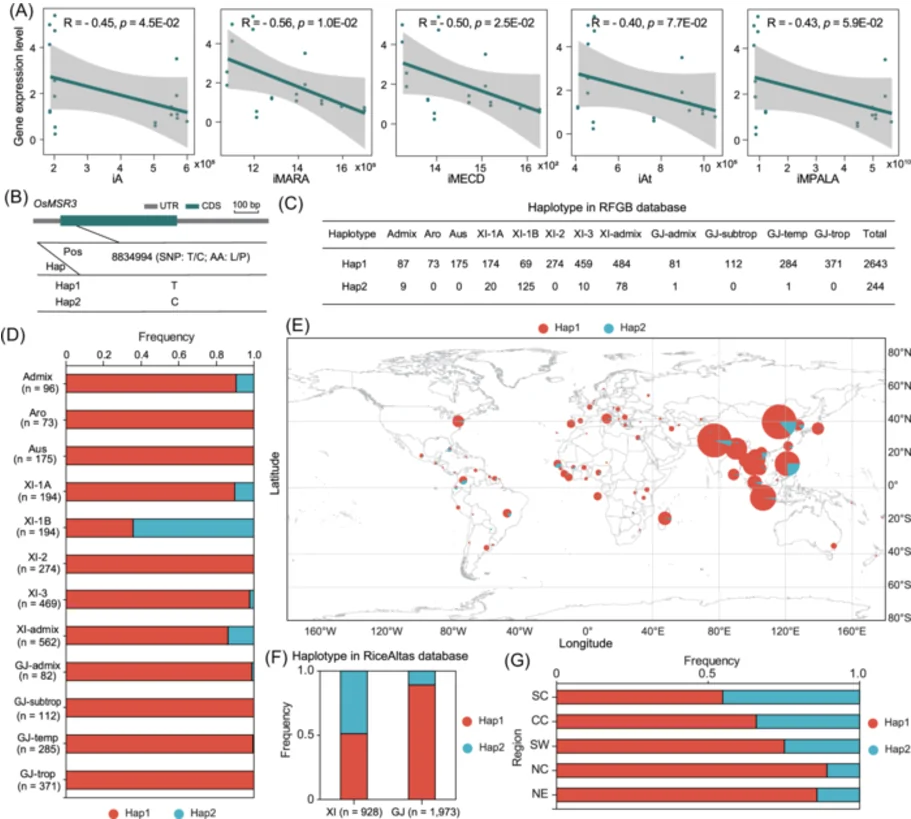
Haplotype analysis of *OsMSR3*. (A) The gene expression level of *OsMSR3* is significantly negatively associated with the image‐based area (iA), min area rectangle area (iMARA), min enclosing circle diameter (iMECD), and associated with the area of top view image (iAt) and second moment principal axis large norm (iMPALA). (B) The SNP (T/C, 50th) in the *OsMSR3* coding DNA sequence (CDS) that results in a missense mutation in amino acid (L/P, 17th), which can divide 2887 rice accessions into two haplotypes (Hap1 and 2). Data were downloaded from the RFGB database, which is described in the method section. (C) The accession number of haplotypes in 12 subpopulations. GJ and XI mean rice subspecies *japonica* and *indica*, respectively. Trop, subtrop, and temp represent tropical, subtropical, and temperate, respectively. (D) Frequency of haplotypes in subpopulations, and the numbers in the left panel mean the total accession number in the corresponding subpopulations. (E) The geographical distribution of haplotypes. These rice accession locations are marked with the capitals of the corresponding countries. (F) The frequency of haplotypes in subspecies XI (*indica*) and GJ (*japonica*) using 2901 rice modern cultivars in China (Data were downloaded from RiceAtlas, as described in the method section). (G) The frequency of haplotypes in five major rice‐growing regions: South China (SC), Central China (CC), Southwest Plateau (SW), North China (NC), and Northeast (NE). Abbreviation: iA, image‐based area; iMARA, min area rectangle area; iMECD, min enclosing circle diameter; iAt, area of top view image (); iMPALA, second moment principal axis large norm; CDS, coding DNA sequence; SNP, single‐nucleotide polymorphism; GJ, *japonica*; XI, *indica*; Trop, tropical; Subtrop, subtropical; Temp, temperate.

We next examined sequence variation within the *OsMSR3* CDS and retained only the non‐synonymous SNPs for haplotype analysis (Table S17). A single SNP (T/C, at position 50), resulting in a missense mutation in the amino acid (L/P, at position 17), was selected for further study (Figure 6B). Among 2887 rice accessions, two haplotypes were identified (Hap1 and Hap2), with Hap1 being the dominant haplotype (2643 accessions) (Figure 6C and Table S17). Of the 244 Hap2 accessions, 233 (95.49%) belonged to the *indica* (XI) subspecies, while only two accessions were from subspecies *japonica* (GJ) (Figure 6C), indicating a strong *indica*‐specific distribution. Analysis across 12 subpopulations further confirmed this subspecies bias, suggesting that this *OsMSR3* mutation may be associated with rice subspecies differentiation (Figure 6D).

Given this pattern, we examined the geographic distribution of OsMSR3 haplotypes (Figure 6E). Hap2 was more prevalent at low latitudes, including Central America and West Africa, and declined progressively toward higher latitudes (Figure 6F). Using RiceAtlas data for modern Chinese rice cultivars, we further assessed haplotype frequencies across five major rice‐growing regions: South China (SC), Central China (CC), Southwest Plateau (SW), North China (NC), and Northeast (NE) (Figure 6F,G and Table S18). Haplotype distributions in both rice subspecies, *indica* and *japonica* cultivars, mirrored trends observed in the 3K dataset, with Hap2 frequency gradually decreasing from South China to Northeast China. This gradient corresponds with the geographic distribution of Cd‐contaminated soils, suggesting that Hap2 may represent a molecular determinant of high‐latitude adaptability and could be valuable for future crop improvement under global warming driven by eCO_2_.

Overall, these findings indicate that a single SNP‐derived haplotype variation in *OsMSR3* may provide a promising target for breeding rice germplasm with reduced Cd accumulation and enhanced adaptation to elevated CO_2_ conditions.

## 3 DISCUSSION

### Constructing an integrative phenotype‐transcriptome network in rice under Cd and eCO_2_ conditions

Although several Cd‐ and eCO_2_‐responsive genes have been identified in rice and their mechanisms partially elucidated [24, 25, 26, 27, 28, 29, 30, 38], the structure of their co‐expression networks remains poorly understood. Such knowledge is essential for improving rice tolerance to Cd under future eCO_2_ conditions, particularly through multi‐target gene editing. In this study, we constructed an integrative phenotype‐transcriptome network by combining multi‐tissue transcriptomics with high‐throughput phenomics under eCO_2_ and Cd stress. We identified five key subnetworks centered on the known Cd‐responsive genes as the hub genes, each exhibiting strong tissue specificity (Figure 5). Several targets were co‐expressed with two or three hub genes, although published transcriptomic datasets are currently insufficient to validate all predicted relationships (Figure 5B,D). These findings demonstrate that our phenotype‐transcriptome network provides a valuable framework for exploring regulatory interactions, while also highlighting the need for further experimental validation.

### High‐throughput phenomics analysis reveals phenotypic similarities and differences in rice responses to Cd and eCO_2_

Coupled with sequencing technologies, high‐throughput phenomics has proven highly effective for functional gene discovery [35, 41]. However, due to experimental constraints, phenomic profiling across key developmental stages under combined eCO_2_ and Cd stress in rice has remained limited. By integrating FACE and HTP systems, we generated comprehensive phenomic data for the *japonica* cultivar Nipponbare across six growth stages under both stresses (Figure 1). Several image‐based traits (i‐traits), including i‐Co, i‐Cot, i‐A, and i‐MARA, exhibited distinct developmental trajectories and differential responses at maturation under eCO_2_ versus Cd stress. Biomass increased significantly under eCO_2_ but decreased under Cd stress. i‐Co followed a similar pattern, whereas i‐MARA displayed the opposite trend relative to biomass. These results highlight both shared and divergent phenotypic responses to Cd and eCO_2_, which differ from those captured by traditional manual measurements. Compared with conventional phenotyping, high‐throughput phenomics enables rapid, precise, and multidimensional characterization of stress responses, facilitating integrative association analyses with genotype data for gene discovery.

### Differentially expressed genes and hormone‐related genes show a similar expression pattern in response to both Cd and eCO_2_

Phytohormones, such as ABA, JA, and SA, are well‐established mediators of Cd stress responses [32, 33, 34], yet the involvement of other hormone classes and the role of hormones under eCO_2_ in rice remains unclear. Previous studies have shown that hormone‐related genes exhibit dynamic expression during node development and embryonic leaf formation in maize [41, 42]. Here, we conducted a comprehensive transcriptomic analysis of genes associated with eight major hormones (Figure 3A−E and Figure S7A−C). Most of the hormone‐related genes exhibited strong tissue specificity, mirroring the patterns observed for DEGs. Notably, 85.64% (9030 of 10,544) of DEGs under Cd stress and 57.64% (15,714 total) under eCO_2_ overlapped (Figure 2G and Table S2), indicating that largely shared transcriptional networks operate under both stresses. These overlapping genes represent valuable resources for investigating rice responses to simultaneous Cd and eCO_2_ exposure. However, our analysis focused on acute contamination; transcriptional responses following soil aging under Cd stress remain unresolved and warrant further investigation. Moreover, the transcriptional regulatory mechanisms in rice subjected to combined eCO_2_ and Cd stress under multi‐chamber conditions remain to be fully elucidated.

### Population analysis of *OsMSR3* identifies a potential breeding target for low‐Cd rice under rising CO_2_

*OsMSR3*, a class I small heat shock protein, is induced by Cd stress, and both transgenic *Arabidopsis* expressing the *OsMSR3* and rice overexpressing this gene exhibit enhanced Cd tolerance [24, 38]. In this study, *OsMSR3* emerged as a hub gene in the yellow module, which was significantly positively associated with the panicle tissues (Figures 4C,D and 5A). We found that *OsMSR3* expression level was significantly negatively correlated with key i‐traits, including i‐A, i‐MARA, and i‐MECD (Figure 6A). Population analyses across two representative rice datasets revealed that the superior allele Hap2 decreased in frequency from low to high latitudes and from South China to Northeast China, mirroring the geographic distribution of Cd‐contaminated soils. This pattern suggests that Hap2 may serve as a valuable target for breeding rice cultivars with reduced Cd accumulation under future eCO_2_ conditions (Figure 6B−G). Previous studies further showed that transgenic *Arabidopsis* expressing *OsMSR3* accumulated higher levels of Cd, phytochelatins (PCs), nonprotein thiol (NPT), and glutathione (GSH) compared with the wild type plants under Cd conditions and accompanied by enhanced expression of Cd‐responsive genes and bHLH (basic helix‐loop‐helix) transcription factors [24], and that *OsMSR3* protects rice seedlings from Cd‐induced damage to photochemical systems and cellular membranes caused by Cd stress [38]. Collectively, these findings indicate that *OsMSR3* mediates Cd stress responses through coordinated physiological and transcriptional regulation, making it a promising target for molecular breeding to improve rice tolerance to Cd and eCO_2_.

In summary, we established a comprehensive transcriptome‐phenomics network governing rice responses to eCO_2_ and Cd stress, characterized by strong tissue specificity. The identified gene modules, hub networks, and superior alleles provide valuable resources for the rational design and breeding of rice cultivars with reduced Cd accumulation.

## 4 CONCLUSION

In this study, we integrated high‐throughput phenomics with multi‐tissue transcriptomics to elucidate the transcriptional regulatory mechanisms underlying rice responses to Cd and eCO_2_ stress. We documented dynamic changes in key i‐traits, such as i‐A and i‐Co, under both stresses, and observed significant increases in tiller number and biomass under eCO_2_ alongside marked reductions under Cd stress. The DEGs and hormone‐related genes displayed dynamic, tissue‐specific expression patterns. We further constructed a co‐expression network and identified hub modules and regulatory subnetworks linked to phenotypic traits, and uncovered a superior *OsMSR3* allele whose frequency declines from low‐ to high‐latitudes. Collectively, these findings reveal a comprehensive transcriptional regulatory framework governing rice responses to Cd and eCO_2_, providing mechanistic insights and valuable genetic resources for breeding rice varieties with low‐Cd accumulation under continuously rising atmospheric CO_2_ levels.

## 5 METHODS

### Experimental site, plant growth, and treatment conditions

The free‐air CO_2_ enrichment (FACE) system and the high‐throughput automatic phenomics platform (HTP) [35] under controlled greenhouse conditions are located at the International Agricultural High‐Tech Industrial Park of the Chinese Academy of Agricultural Sciences in Langfang City, Hebei Province, China (39°36'51.005'' N, 116°36'31.068'' E). The site has a warm temperate continental monsoon climate, a mean annual temperature of 11.9°C, average annual precipitation of 554.9 mm, and a frost‐free period of approximately 183 days. In this study, the FACE experiments included three elevated CO_2_ (eCO_2_) treatments, and each was conducted in a circular greenhouse (5 m^2^) spaced > 5 m apart to avoid CO_2_ cross‐contamination. The eCO_2_ treatments received additional concentrations of 600, 1200, and 1800 μmol mol^−1^ CO_2_ above ambient (~400 μmol mol^−1^), hereafter referred to as C6, C12, and C18, respectively. The pure CO_2_ was delivered to the greenhouse centers via the FACE system, and enrichment was maintained continuously from seedling establishment through harvest. To minimize positional bias arising from CO_2_ gradients, plants were transported to the HTP for imaging and then returned to the FACE system, where they were randomly repositioned after data collection. For the Cd treatment, the rice plants were irrigated with 2 L of 7.5 mg L^−1^ CdCl_2_ solution at the seedling growth stage (refer to the Cd concentration set in this study [13]). The control group (hereafter referred to as CK) was grown under ambient CO_2_ and Cd‐free conditions.

The *japonica* rice cultivar Nipponbare (hereafter referred to as Nip) was used as the experimental material. Seeds were germinated and sown in the seedling trays, and the 30‐day‐old seedlings were transplanted into pots (25 cm diameter) containing 6 kg of nutrient soil composed of a high‐quality shallow peat‐based substrate (PINDSTRUP Seeding; 5.5 < pH ≤ 6.0, EC = 20 mS m^−1^, fertilizer dosage = 0.55 kg m^−3^, and extremely low levels of pollutants (such as Cd). The main components of the fertilizer include Nitrogen (39 gr m^−3^), Ammonium (28 gr m^−3^), Phosphorus (P: 33 gr m^−3^ and P_2_O_5_: 76 gr m^−3^), Potassium (K: 110 gr m^−3^ and K_2_O: 132 gr m^−3^), and other trace elements, such as Iron (0.7 gr m^−3^) and Zinc (2.1 gr m^−3^), etc. Detailed fertilizer specifications are available on the pindstrup website. After 10 days of growth, plants assigned to eCO_2_ treatments were transferred to the FACE system, and Cd‐treated plants received 2 L of CdCl_2_ solution applied uniformly to each pot. Each treatment group included 18 rice plants that were divided into three biological replicates, with six plants per group selected for the acquisition of high‐throughput phenotyping from the seedling growth stage to the maturation stage. Experiments were conducted from May to October 2021. All rice plants were irrigated with 2 L of water every 2 days until maturity, with no supplemental fertilization.

### High‐throughput phenotypic image acquisition and trait extraction

To capture detailed and dynamic phenotypes of rice under control and treatment conditions, we acquired high‐throughput phenotypic images weekly using the HTP system [35] spanning the entire growth period from seedling to maturity. The HTP comprised eight transport pipelines, each with 57 conveyors, accommodating up to 456 pots simultaneously for growth and imaging. A Basler piA 2400 camera (Basler AG) equipped with a 2/300 CCD progressive scan w sensor generated images at a resolution of 2456 × 2058 pixels with a pixel size of 3.45 × 3.45 mm.

For each plant, six images were captured from front, side, and top perspectives, requiring approximately 40 s per plant. Image analysis followed methods described previously [35], which was carried out using LemnaGrid software that was developed by LnmnaTec to address phenotyping extraction. Briefly, images were reconstructed using a Bayer color pattern, converted to binary format, and segmented to extract plant skeletons and regions. Quantitative traits were then computed from the digital images. A total of 54 image‐based traits (i‐traits), including area (i‐A), minimum area rectangle area (i‐MARA), minimum enclosing circle diameter (i‐MECD), and compactness (i‐Co) from front‐view images, as well as compactness top (i‐Co) and area top (i‐At) from the top‐view images, were extracted based on the image analysis system of the HTP [35]. The trait definitions and calculation methods of i‐traits were described in our previous publication [35] and are available at the GitHub repository with the link: https://github.com/GUOWEIJUN/Rice_integrative_network_under_Cd_and_eCO2. Statistically significant differences in i‐traits among treatments were assessed using the Kruskal‐Wallis test.

### Sampling

Six developmental stages were selected for the next analysis according to Zadoks' decimal growth scale (https://agriculture.vic.gov.au/crops-and-horticulture/grains-pulses-and-cereals/crop-production/general-agronomy/decimal-growth-scale-of-cereals): seedling growth, tillering, elongation, booting, flowering, and maturation stages (S1 − S6). The rice plants are at two key growth and development stages (the elongation stage (S3; ES) and maturation stage (S6; MS)). The leaves and stems in both ES and MS, and the young panicles at MS from six plants, were mixed as one replicate; a total of two independent biological replicates were collected and stored after quick‐freezing using liquid nitrogen for the next RNA extraction and transcriptomic sequencing. Detailed transcriptome information is provided in Table S1.

### RNA isolation, library construction, and sequencing

Total RNA was extracted from the samples using the RNeasy Plus Mini Kit (Vazyme) according to the manufacturer's protocol. The integrity and quality of RNA were assessed using an RNA 6000 Nano Kit on an Agilent 2100 Bioanalyzer System. The cDNA libraries were constructed with a NEBNext Ultra RNA Library Prep Kit (NEB) and sequenced on the Illumina HiSeq X‐ten platform.

### RNA‐seq data analysis

The RNA‐seq analysis pipeline followed a previously published protocol [41]. Briefly, the low‐quality reads and adapters were removed, and the clean reads were aligned to the rice reference genome sequences using Hisat2 with default parameters [43]. Only the uniquely mapped reads were retained for the next gene expression quantification using the featureCounts software [44], and expression levels were normalized as the FPKM (Fragments Per Kilobase Million) value. Differential expression genes (DEGs) were determined between the treatment and control groups using the DESeq2 package [45] in R software. Genes with fold change (FC) ≥ 2 and FDR < 0.05 were considered as DEGs. Gene ontology (GO) enrichment analysis of DEGs was performed using the online tools agriGO v2.0 (https://systemsbiology.cau.edu.cn/agriGOv2/index.php/manual.php) with default parameters, and the terms with FDR < 0.05 were considered significantly enriched.

### Expression pattern analysis of major phytohormones

The hormone‐related genes were obtained from the MapMan database (https://mapman.gabipd.org/mapmanstore). Eight major phytohormones‐auxin, salicylic acid (SA), ethylene, abscisic acid (ABA), brassinolide (BR), cytokinin (CK), jasmonic acid (JA), and gibberellic acid (GA)‐were classified into three functional categories, namely synthesis degradation (C1), signal transduction (C2), and induced regulated responsive activated (C3). Detailed gene annotations are provided in Tables S4−S11. The heatmaps of hormone gene expression level were generated using the pheatmap package in R software.

### Construction of co‐expression networks

To characterize gene‐gene relationships across 50 samples, a gene expression matrix (35,678 × 50) was used to construct a weighted gene co‐expression network using the WGCNA package [46] with a soft‐thresholding power (*β*) of 17, minimum module size of 100, and branch merge cut height of 0.25 in R software (https://www.r-project.org/). A total of 31 gene modules were identified using the dynamic cut tree. Module‐trait correlations were then calculated, and modules showing the highest correlations (*p*‐value < 0.05) were selected for subnetwork construction and hub gene identification. The networks were visualized using Cytoscape v3.6.1 software (http://cytoscape.org/), based on node and edge information.

### Haplotype analysis of *OsMSR3*

The high‐quality SNPs within the *OsMSR3* gene (*LOC_Os03g16030*) coding DNA sequence (CDS) were retrieved from the RFGB Database (https://v1.rmbreeding.cn/) and analyzed using the RFGB tools (https://v1.rmbreeding.cn/Index/). SNP functional effects were predicted using the online Variant Effect Predictor (https://ensembl.gramene.org/Oryza_sativa/Tools/VEP). Haplotype frequencies were calculated for each rice subpopulation (defined by the 3K Rice Genomes Project) [39], and geographic distributions were inferred using accession location data. The geographic distributions were conducted in the online web tools (https://www.bioinformatics.com.cn/), which map information was provided by the Institute of Geographic Sciences and Natural Resources Research of the Chinese Academy of Sciences. In addition, haplotype frequencies were analyzed across rice subspecies XI (*indica*) and GJ (*japonica*) and five major rice‐growing regions‐South China (SC), Central China (CC), Southwest Plateau (SW), North China (NC), and Northeast (NE)‐using the RiceAtlas database, which contains 2901 modern Chinese rice cultivars [40]. Detailed information of haplotypes is provided in Tables S17 and S18.

### Statistical analysis and data visualization

All statistical analyses and Pearson correlation tests were performed in R version 3.5.1 software (https://www.r-project.org/). Group comparisons were conducted using the Kruskal–Wallis test for multiple groups and the paired Student's *t*‐tests where appropriate. Detailed descriptions of statistical procedures are provided in the figure legends. The *p*‐value < 0.05 was considered statistically significant for all analyses.

## AUTHOR CONTRIBUTIONS

Li Pu, Xiaofeng Gu, Jian Tian, and Weixuan Wang designed and supervised the project. Weijun Guo and Hanlin Liu performed the trials and collected the high‐throughput phenomics, data analysis, network construction, and haplotype analysis. Dongwei Li, Shang Xie, Yichao Mao, Liwen Yang, and Cong Li prepared the RNA sequencing. Weijun Guo wrote the manuscript. Li Pu and Xiaofeng Gu revised the manuscript. All authors have read the final manuscript and approved it for publication.

## CONFLICT OF INTEREST STATEMENT

The authors declare no conflicts of interest.

## ETHICS STATEMENT

No animals or humans were involved in this study.

## Supporting information


**Figure S1:** Three key i‐traits of phenimics in response to elevated CO_2_ concentration and cadmium in rice.
**Figure S2:** The correlation between biological replicates of the multi‐tissue transcriptom.
**Figure S3:** Dynamics change of DEGs under three eCO2 concentration levels.
**Figure S4:** Gene ontology (GO) enrichment analysis of DEG in leaf and stem samples at the elongation stage under eCO_2_ conditions.
**Figure S5:** Gene ontology (GO) enrichment analysis of DEG in leaf, stem, and young panicle samples at the maturation stage under eCO_2_ conditions.
**Figure S6:** Gene ontology (GO) enrichment analysis of DEG in leaf, stem, and young panicle samples under Cd conditions.
**Figure S7:** Heatmap of hormone‐related genes.
**Figure S8:** Gene ontology (GO) enrichment analysis of genes in four modules.

**Table S1:** The detailed information of the transcriptome.
**Table S2:** The 1540 overlapped genes between Cd and eCO_2_ conditions.
**Table S3:** The Gene Ontology (GO) results for the overlapping genes.
**Table S4:** The information and expression level of auxinrelated genes.
**Table S5:** The information and expression level of salicylic acid (SA)‐related genes.
**Table S6:** The information and expression level of ethylene‐related genes.
**Table S7:** The information and expression level of abscisic acid (ABS)‐related genes.
**Table S8:** The information and expression level of brassinosteroid (BR)‐related genes.
**Table S9:** The information and expression level of cytokinin (CK)‐related genes.
**Table S10:** The information and expression level of gibberellic acid (GA)‐related genes.
**Table S11:** The information and expression level of jasmonate (JA)‐related genes.
**Table S12:** The top 20 significantly enriched GO terms in four modules.
**Table S13:** The subnetwork information of the yellow module.
**Table S14:** The subnetwork information of the blue module.
**Table S15:** The subnetwork information of the dark‐red module.
**Table S16:** The subnetwork information of the turquoise module.
**Table S17:** The haplotype information of *OsMSR3*.
**Table S18:** The haplotype proportion of *OsMSR3* using RiceAtlas data.

## Data Availability

The raw data of transcriptome have been deposited in the Genome Sequence Archive [47] in the National Genomics Data Center under BioProject PRJCA029793 (https://ngdc.cncb.ac.cn/bioproject/browse/PRJCA029793) and are accessible with the GSA numbers CRA021257 (https://ngdc.cncb.ac.cn/gsa/search?searchTerm=CRA021257) and CRA018773 (https://ngdc.cncb.ac.cn/gsa/search?searchTerm=CRA018773). Analysis pipeline codes were uploaded to the GitHub repository (https://github.com/GUOWEIJUN/Rice_integrative_network_under_Cd_and_eCO2). Supplementary materials (figures, tables, graphical abstract, slides, videos, Chinese translated version, and updated materials) may be found in the online DOI or iMetaOmics Science http://www.imeta.science/imetaomics. The data that supports the findings of this study are available in the supplementary material of this article.
