# Supplementary material for: Integrated high‐throughput phenomics and transcriptomics uncover the transcriptional mechanisms underlying rice responses to elevated CO2 concentration and cadmium stress
Source: IMetaOmics. 2026 May 20;3(2):e70100. doi: 10.1002/imo2.70100 (PMC13674439; doi:10.1002/imo2.70100)
Supplement: Supplementary file 1 [file IMO2-3-e70100-s002.docx]

Supporting Information to

**Integrated high-throughput phenomics and transcriptomics uncover the transcriptional mechanisms underlying rice responses to** **elevated CO_2_ concentration and cadmium stress**

**Running title:** An integrative transcriptional regulatory network in rice under eCO_2_ and Cd stress

Weijun Guo^1#^, Hanlin Liu^1#^, Dongwei Li^1^, Shang Xie^1^, Yichao Mao^1^, Liwen Yang^1^, Cong Li^1^, Weixuan Wang^1*^, Jian Tian^2*^, Xiaofeng Gu^1*^, Li Pu^1*^

^1^Biotechnology Research Institute, Chinese Academy of Agricultural Science, Beijing 100081, China

^2^Institute of Animal Science, Chinese Academy of Agricultural Science, Beijing 100081, China

^#^These authors contributed equally: Weijun Guo, Hanlin Liu

^*^Correspondence: [puli@caas.cn](mailto:puli@caas.cn) (Li Pu), [guxiaofeng@caas.cn](mailto:guxiaofeng@caas.cn) (Xiaofeng Gu), [tianjian@caas.cn](mailto:tianjian@caas.cn) (Jian Tian), [wangweixuan@caas.cn](mailto:wangweixuan@caas.cn) (Weixuan Wang)

**Figure S1 Three key i-traits of phenimics in response to elevated CO_2_ concentration and cadmium in rice.** (A−C) The image-based area top (i-At (A)), image-based second moment principal axis large norm (i-MAPLA (B)), and image-based min enclosing circle diameter (i-MECD (C)) at six representative growth stages under eCO_2_ and Cd stress. Statistical significances were determined by the Kruskal-Wallis test.

**Figure S2 The correlation between biological replicates of the multi-tissue transcriptome.** L, S, and P represent leaf, stem, and young panicle samples, respectively. The eCO_2_ groups were treated with an additional 600, 1200, and 1800 μmol mol^-1^ CO_2_ (hereafter referred to as C6, C12, and C18, respectively) more than the control group. ES and MS represent the elongation stage and the maturation stage. Abbreviation: PCC, Pearson correlation coefficient.

**Figure S3 Dynamics change of DEGs under three eCO_2_ concentration levels.** (A) K-means analysis of 17,947 differentially expressed genes under three elevated carbon dioxide (eCO_2_) concentrations. Gene expression levels were transformed using the *Z*-score. (B) Transcription factor enrichment analysis for five cluster genes. (C) Gene Ontology enrichment analysis of five cluster genes. The top 10 significantly enriched terms of each cluster were selected, and the significance was transformed using the -log_10_(FDR) value.

**Figure S4 Gene ontology (GO) enrichment analysis of DEG in leaf and stem samples at the elongation stage under eCO_2_ conditions.** (A−C) The results of leaf samples. (D−F) The results of stem samples. Abbreviation: FDR, false discovery rate.

**Figure S5 Gene ontology (GO) enrichment analysis of DEG in leaf, stem, and young panicle samples at the maturation stage under eCO_2_ conditions.** (A−C) The results of leaf samples. (D−F) The results of stem samples. (G−I) The results of young panicle samples. Abbreviation: FDR, false discovery rate.

**Figure S6 Gene ontology (GO) enrichment analysis of DEG in leaf, stem, and young panicle samples under Cd conditions.** (A and B) GO results of samples at the elongation stage. (C−E) The GO results of samples at the maturation stage. Abbreviation: FDR, false discovery rate.

**Figure S7 Heatmap of hormone-related genes.** (A−C) The normalized gene expression levels of salicylic acid (SA) (A), cytokinin (CK) (B), and gibberellic acid (GA) are shown. These genes are divided into three functional categories: synthesis and degradation (C1), (C2) signal transduction, and (C3) induced regulated responsive activated. Dotted rectangles represent these genes expressed in a tissue-specific manner. Abbreviation: SA, salicylic acid; CK, cytokinin; GA, gibberellic acid.

**Figure S8 Gene ontology (GO) enrichment analysis of genes in four modules.** (A−D) Top 20 significantly enriched terms (FDR < 0.05) in blue (A), yellow (B), turquoise (C), and darkred (D) modules. Only seven terms were significantly enriched in the darkred module.
